# Clinical Features of Pediatric Idiopathic Intracranial Hypertension and Applicability of New ICHD-3 Criteria

**DOI:** 10.3389/fneur.2018.00819

**Published:** 2018-09-28

**Authors:** Romina Moavero, Giorgia Sforza, Laura Papetti, Barbara Battan, Samuela Tarantino, Federico Vigevano, Massimiliano Valeriani

**Affiliations:** ^1^Child Neurology Unit, Department of Neuroscience and Neurorehabilitation, Headache Center, Bambino Gesù Children's Hospital, IRCCS, Rome, Italy; ^2^Child Neurology and Psychiatry Unit, Tor Vergata University Hospital of Rome, Rome, Italy; ^3^Center for Sensory Motor Interaction, Aalborg University, Aalborg, Denmark

**Keywords:** pseudotumor cerebri, idiopathic intracranial hypertension, papilloedema, children, adolescents, ICHD-2, ICHD-3

## Abstract

Idiopathic intracranial hypertension (IIH) is characterized by intracranial pressure >28 cmH_2_O in the absence of identifiable causes. Aim of this paper is to describe the clinical phenotype of pediatric IIH and to analyze the applicability of ICHD-3 criteria in comparison to the ICHD-2. We conducted a retrospective analysis of full clinical data of pediatric patients diagnosed with IIH between January 2007 and June 2018. Diagnostic evaluation included neuroimaging (all patients) and ultrasound-based optic nerve sheath diameter measurement (9 patients). Diagnosis of IIH was verified according to both ICHD-2 and ICHD-3 criteria for headache attributed to IIH, to verify the degree of concordance. We identified 41 subjects with suspected IIH; 14 were excluded due a diagnosis of secondary IH or lack of data. We therefore selected 27 subjects (age 4–15 years, mean 11). All patients presented with headache and bilateral papilloedema. Headache was daily in 22% cases, with diffuse gravative pain in 41%. In 4%, pain was exacerbated by cough, stress or tension. The most common presentation symptoms, in addition to headache, were blurred vision or diplopia (70%), vomiting (33%), and dizziness (15%). Twenty patients (74%) were obese. In 6 patients (22%) neuroimaging showed empty sella. Optic nerve sheath distension was detected in 6 out of 9 patients. Regarding the applicability of the ICHD-2 criteria, 18/27 (71%) patients have criterion A; 24/27 (89%) criterion B; 27/27 (100%) criterion C; 27/27 (100%) criterion D. When the ICHD-3 criteria were used, 27/27 (100%) fitted criterion A; 24/27 (89%) criterion B; 27/27 (100%) criterion C; and 27/27 (100%) criterion D. Our study suggests that, as compared with the ICHD-2, the new ICHD-3 criteria for headache attributed to IIH are better satisfied by pediatric patients with IIH. This is mainly due to the fact that qualitative headache characteristics are no longer considered in ICHD-3. Although the risk of under-rating the symptom of headache in IIH should not be disregarded, in pediatric population headache characteristics are usually less defined than in adults and obtaining a precise description of them is often very difficult.

## Introduction

Idiopathic intracranial hypertension (IIH), also known as pseudotumor cerebri syndrome (PTC), is a rare pediatric neurological disorder (1). It is characterized by increased intracranial pressure (ICP) in the absence of any brain parenchymal lesions, vascular malformations, hydrocephalus, or central nervous system (CNS) infection (2). The diagnosis is usually confirmed by high opening pressure (OP) of cerebrospinal liquid (CSF) (more than 25 cm H_2_O), provided there are no secondary causes of intracranial hypertension. In 2013, the opening pressure (OP) for children aged from 1 to 18 years of age was redefined, and the upper limit for a normal OP is actually 28 cm H_2_O in the pediatric population (1, 3).

IIH is more frequent in females (females-males ratio 4:1), especially in the reproductive age, with overweight being a significant adjunctive risk factor. Indeed, in fertile age overweight females, the estimated incidence is 12–20 per 100,000 people per year, vs. a general incidence in the adult population of 0.5–2 per 100,000 (4, 5). The exact prevalence of IIH in the pediatric population is not yet well established. Recently, studies from the United Kingdom applying the Friedman criteria revealed an annual incidence of 0.71 per 100,000 (1, 6).

The pathogenesis of IIH is still largely unknown. ICP is determined by the balance between production and absorption of CSF. According to the Monro-Kellie rule, an increase in ICP might be related to increased CSF, expanded brain tissue, or increased blood volume (7). Proposed hypotheses include excess of CSF production, CSF outflow reduction, increase in cerebral blood volume and/or brain water content, obstruction to venous system, endocrinological or metabolic causes, chronic inflammation, and obesity (in pre- and post-pubertal females) (8–15).

The characteristic signs and symptoms of IIH were initially described by Dandy and were later organized into the Modified Dandy Criteria by Smith, combining the lack of other causes of increased ICP (such as neoplasms and cerebral venous sinus thrombosis—CVST), with the presence of the following features: symptoms of increased ICP, papilloedema and raised opening cerebrospinal fluid (CSF) pressure at lumbar puncture (LP) (16) (see Table 1). In 2013, revised diagnostic criteria for IIH have been published by Friedman and coworkers, not including symptoms of raised ICP (Table 2) (3). According to these revised criteria, IIH can be classified as “definite” (increased OP and either papilloedema or abducens nerve palsy), “probable” (normal CSF pressure in presence of papilloedema), or “suggestive of” (raised CSF pressure plus at least three valid neuroimaging markers of raised ICP, in the absence of papilloedema and abducens nerve palsy) (3).

**Table 1:** Modified Dandy Criteria (16).

1. Signs and symptoms of increased intracranial pressure (headaches, nausea, vomiting, transient obscurations of vision, papilledema). 2. No localizing neurologic signs otherwise, with the single exception being unilateral or bilateral VI nerve paresis. 3. CSF can show increased pressure, but no cytologic, or chemical abnormalities otherwise. 4. Normal to small symmetric ventricles must be demonstrated (originally required ventriculography, but now demonstrated by CT).

**Table 2:** Diagnostic criteria for pseudotumor cerebri syndrome.

1. Required for diagnosis of pseudotumor cerebri syndrome^a^
Papilledema Normal neurologic examination except for cranial nerve abnormalities Neuroimaging: Normal brain parenchyma without evidence of hydrocephalus, mass, or structural lesion and no abnormal meningeal enhancement on MRI, with and without gadolinium, for typical patients (female and obese), and MRI, with and without gadolinium, and magnetic resonance venography for others; if MRI is unavailable or contraindicated, contrast-enhanced CT may be used Normal CSF composition Elevated lumbar puncture opening pressure [≥250 mm CSF in adults and ≥280 mm CSF in children (250 mm CSF if the child is not sedated and not obese)] in a properly performed lumbar puncture
2. Diagnosis of pseudotumor cerebri syndrome without papilledema
In the absence of papilledema, a diagnosis of pseudotumor cerebri syndrome can be made if B–E from above are satisfied, and in addition the patient has a unilateral or bilateral abducens nerve palsy
In the absence of papilledema or sixth nerve palsy, a diagnosis of pseudotumor cerebri syndrome can be suggested but not made if B–E from above are satisfied, and in addition at least 3 of the following neuroimaging criteria are satisfied: Empty sella Flattening of the posterior aspect of the globe Distention of the perioptic subarachnoid space with or without a tortuous optic nerve Transverse venous sinus stenosis

a *A diagnosis of pseudotumor cerebri syndrome is definite if the patient fulfills criteria A–E. The diagnosis is considered probable if criteria A–D are met but the measured CSF pressure is lower than specified for a definite diagnosis*.

Headache is the most common presentation symptom of IIH. However, the characteristics of the headache in IIH patients are widely variable and not specific to IIH. Headache is often referred as unusually severe and can be lateralized and throbbing or pulsatile. It can be intermittent or persistent, occurring daily or less frequently, and nausea and vomiting can be present. Headache can be exacerbated by posture changes, and some patients may report relief with non-steroidal anti-inflammatory drugs and/or rest, although drug refractoriness is common. Therefore, in most cases, headache characteristics are similar to migraine and tension-type headache (17). If retrobulbar pain and pain with eye movement or globe compression are present, they can be highly suggestive of IIH (17). Since headache may be the main symptom of changes in ICP, diagnostic criteria for “Headache attributed to IIH” have been published by the Headache Classification Committee of the International Headache Society (IHS) in the second international classification of migraine disorders in 2004 and subsequently modified in 2018 in the third classification (18, 19) (Tables 3, 4). Some patients, especially younger children, might present intracranial hypertension without headache (20). In the absence of headache, the diagnosis is often suggested by accidental finding of papilloedema during routine ophthalmologic evaluations.

**Table 3:** ICHD-2 criteria for headache attributed to IIH (19).

**Headache attributed to IIH**
(A) Progressive headache with at least one of the following characteristics and fulfilling criteria C and D: Daily occurrence Diffuse and/or constant (non-pulsating) pain Aggravated by coughing or straining
(B) Intracranial hypertension fulfilling the following criteria: Alert patient with neurological examination that either is normal or demonstrates any of the following abnormalities: Papilloedema Enlarged blind spot Visual field defect (progressive if untreated) Sixth nerve palsy Increased CSF pressure (200 mm H_2_O in the non-obese, 250 mm H_2_O in the obese) measured by lumbar puncture in the recumbent position or by epidural or intraventricular pressure monitoring Normal CSF chemistry (low CSF protein is acceptable) and cellularity Intracranial diseases (including venous sinus thrombosis) ruled out by appropriate investigations No metabolic, toxic or hormonal cause of intracranial hypertension
(C) Headache develops in close temporal relation to increased intracranial pressure
(D) Headache improves after withdrawal of CSF to reduce pressure to 120–170 mm H_2_O and resolves within 72 h of persistent normalization of intracranial pressure

**Table 4:** ICHD-3 criteria for headache attributed to IIH (18).

**Headache attributed to IIH**
(A) New headache, or a significant worsening of a pre-existing headache, fulfilling criterion C
(B) Both of the following: Idiopathic intracranial hypertension (IIH) has been diagnosed cerebrospinal fluid (CSF) pressure exceeds 250 mm CSF (or 280 mm CSF in obese children)
(C) Either or both of the following: Headache has developed or significantly worsened in temporal relation to IIH, or led to its discovery Headache is accompanied by either or both of following: pulsatile tinnitus papilloedema
(D) Not better accounted for by another ICHD-3 diagnosis

## Objective of the study

The aim of this study was to analyze the applicability of the new ICHD-3 criteria in comparison to the ICHD-2 criteria in a sample of pediatric patients suffering from headache attributed to IIH.

## Patients and methods

We retrospectively analyzed clinical, laboratory, and neuroimaging data of pediatric patients admitted for headache and finally diagnosed with IIH in the Headache Center of Bambino Gesù Children's Hospital between January 2007 and June 2018. Patients with incomplete data for whom a complete verification of diagnosis was not possible were not included in the present study. Also patients with secondary IH have been excluded. Diagnostic evaluation included neuroimaging studies for all patients, and in some cases ultrasound-based optic nerve sheath diameter (ONSD) measurement. All patients/parents/caregivers have been contacted by phone to evaluate whether they suffered from headache before the acute episode, and to understand if clinical characteristics presented some differences.

In all patients diagnosis of IIH was verified both according to ICHD-2 and ICHD-3 criteria for headache attributed to IIH, to verify the degree of concordance.

## Results

We identified a total of 41 subjects diagnosed with IIH. Four patients have been excluded due to the identification of possible causes of IH (including cyclosporin therapy and hypoparathyroidism); 10 patients received a clinical diagnosis based on the presence of headache, papilloedema, and obesity, but have been excluded due to lack of data (lumbar puncture not performed or data not available). We therefore selected 27 subjects (15 F, 12 M), ranging between 4 and 15 years of age (mean age 11 years). All patients presented with headache and physical examination revealed the presence of bilateral papilloedema in all of them. Only 3 of them (patients #3, #10, #19) reported a previous history of headache, but in all of them pain characteristics were quite different with more severe symptoms and unsatisfactory response to analgesic treatment. Despite clinical features were highly suggestive for IIH in all patients, in 3 of them the opening pressure of CSF was < 25 cmH_2_O thus not satisfying all criteria for the diagnosis of IIH. As for headache characteristics, it was daily in 22% of cases, with diffuse gravative pain in 41% of cases, while throbbing headache was present in 15% of patients. Moreover, in 4% of patients, headache was exacerbated by cough, stress or tension, and in 11% it had a unilateral distribution. The most common presentation symptoms, in addition to headache, were blurred vision or diplopia (70%), vomiting (33%), and dizziness (15%). Twenty patients (74%) were obese (weight centile ≥ 90%).

In the majority of patients neuroimaging was normal, while in 6 patients (22%), MRI or CT showed signs of empty sella syndrome. Ultrasound-based ONSD measurement was obtained only in 9 patients, in six of whom an optic nerve sheath distension could be demonstrated. Clinical characteristics of our sample are summarized in Table 5.

**Table 5:** Clinical characteristics of patients.

**N**	**Age**	**Headache**	**Dizziness**	**Vomiting**	**Papilloedema**	**Visual disturbances**	**Obesity/BMI**	**Opening pressure**	**Neuroimaging**	**ONSD**
1	6y10m	y	n	n	y	n	y/30,2	30 cmH_2_O	Normal	np
2	11y	y	n	n	y	y	y/31,1	29.9 cmH_2_O	Normal	n
3	11y7m	y	n	n	y	y	y/32	40 cmH_2_O	Normal	y
4	12y2m	y	y	y	y	y	y/31,6	71 cmH_2_O	Normal	y
5	13y	y	n	n	y	y	y/33,2	36 cmH_2_O	Normal	y
6	11y	y	n	n	y	y	y/30,9	53 cmH_2_O	Normal	np
7	9y	y	y	n	y	y	Y/33	38 cmH_2_O	Normal	np
8	15y	y	y	n	y	n	y/32	40 cmH_2_O	Normal	np
9	9y	y	n	y	y	y	y/35,2	40.7 cmH_2_O	Normal	n
10	10y	y	n	y	y	y	N/22,3	40 cmH_2_O	Empty sella	np
11	12y	y	n	n	y	y	n/19	44 cmH_2_O	Normal	np
12	14y8m	y	n	y	y	y	n/16,7	65 cmH_2_O	Normal	np
13	11y9m	y	n	n	y	n	y/33,4	55.7 cmH_2_O	Normal	np
14	11y2m	y	n	y	y	y	y/32,1	89 cmH_2_O	Normal	np
15	13y7m	y	n	y	y	y	Y/30,2	47.5 cmH_2_O	Normal	np
16	12y	y	n	n	y	y	y/36,3	54 cmH_2_O	Normal	np
17	15y4m	y	n	n	y	y	y/37,2	48 cmH_2_O	Empty sella	n
18	7y4m	y	n	n	y	y	y/33,3	45 cmH_2_O	Empty sella	np
19	11y11m	y	n	n	y	n	y/57,7	29 cmH_2_O	Normal	np
20	12y11m	y	n	n	y	y	n/18,9	35 cmH_2_O	Normal	y
21	9y3m	y	y	n	y	y	n/19,8	28 cmH_2_O	Normal	np
22	4y8m	y	n	y	y	n	n/19	37 cmH_2_O	Normal	y
23	9y1m	y	n	y	y	n	y/34,1	36 cmH_2_O	Normal	np
24	11y	y	n	n	y	n	n/21,3	50 cmH_2_O	Empty sella	np
25	12y	y	n	n	y	n	y/33,6	21 cmH_2_O	Normal	y
26	9y9m	y	n	n	y	y	y/35,3	20 cmH_2_O	Empty sella	np
27	10y	y	n	y	y	y	y/39	24 cmH_2_O	Empty sella	np

*y, yes; n, no; np, not performed; ONSD, optic nerve sheath diameter*.

Regarding the applicability of the ICHD-2 criteria, 18/27 (71%) patients have criterion A; 24/27 (89%) criterion B; 27/27 (100%) criterion C; 27/27 (100%) criterion D. When ICHD-3 criteria were used, 27/27 patients (100%) fitted criterion A; 24/27 (89%) criterion B; 27/27 (100%) criterion C; and 27/27 (100%) criterion D.

## Discussion

To the best of our knowledge, this is the first study comparing the applicability of the new ICHD-3 criteria to pediatric headache attributed to IIH. According to the results obtained by our study, the new ICHD-3 criteria seem to be applicable and valid for a higher rate of subjects clinically presenting with symptoms suggestive of IIH. In particular, in our clinical series, the difference was evident in the criterion A, which was fulfilled by all patients when applying ICHD3, but only by 71% when considering the old ICHD2 version. Certainly, this is due to the disappearance of specificity criteria for the headache, which now is no longer required to be daily, diffuse, and/or aggravated by cough or straining. Although this might be considered as a worse reliability and as a risk of under-rating headache as a symptom in IIH (21), it should be underlined that in pediatric population headache characteristics are usually less defined than in adults. Moreover, in younger children obtaining a precise description of headache characteristics could be very difficult. In the ICHD-3, it is only requested that headache must be “new” or show a “significant worsening.” This latter expression, according to ICHD-3 specifications, implies at least a double increase in frequency and/or severity of headache. An objective evaluation of a significant worsening was present in all the 3 patients presenting a previous history of migraine, however it could be difficult to be ascertained in younger patients. The ICHD-3 criterion C states that the new or worsened headache leads to the discovery of IIH, thus helping in evaluating the fulfillment of this criterion. Summarizing, the significant change in criterion A with the abolition of precise qualitative characteristics defining headache could help to include a higher rate of pediatric patients presenting with signs and symptoms suggestive of IIH. Another important difference between ICHD versions 2 and 3 is represented by the increase of ICP cut-off in criterion B, keeping a difference between patients with and without obesity. However, in our sample, although limited, this modification did not determine any difference.

A main feature of the ICHD-2 diagnostic criteria was relief from headache after CSF withdrawal, but this was removed in the recently published ICHD-3 criteria. In our series, all patients had an improvement in symptoms after lumbar puncture and CSF withdrawal, but this can be seen also in patients with other types of headache (presenting a sensitivity and specificity of 72 and 77%, respectively). This criterion has been replaced by the criterion according to which headache should be “no better accounted for by another ICHD-3 diagnosis.” This appeared to be a necessary specification since headache secondary to IIH can be really overlapping to chronic migraine or chronic tension-type headache. Indeed, these disorders often coexist with IIH, and in all patients still complaining of headaches after treatment, migraine should be considered in order to use appropriate treatment and prevent unnecessary overtreatment for suspected IIH relapses (22).

## Ethics statement

This retrospective study received approval by the Local Ethical Board of Bambino Ges Children's Hospital, IRCCS, Rome, Italy.

## Author contributions

RM and MV conceived and wrote the paper. MV, GS, LP, BB, and ST collected the data. RM and GS analyzed the data. FV and MV revised the paper.

### Conflict of interest statement

The authors declare that the research was conducted in the absence of any commercial or financial relationships that could be construed as a potential conflict of interest.
